# Intra-thoracic Symptomatic Gallstones in a Right-Sided Post-traumatic Diaphragmatic Hernia: A Case Report

**DOI:** 10.7759/cureus.32824

**Published:** 2022-12-22

**Authors:** Faiza H Soomro, Afnan Hassan, Izza Nazir, Sufyan Azam, Amber Yasmin

**Affiliations:** 1 General Surgery, The Dudley Group NHS Foundation Trust, Dudley, GBR; 2 Hepatobiliary and Pancreatic (HPB) Surgery, King's College Hospital, London, GBR; 3 Internal Medicine, Shifa International Hospital Islamabad, Islamabad, PAK; 4 General Surgery, Shifa International Hospital Islamabad, Islamabad, PAK; 5 Surgery, Queen Elizabeth Hospital Birmingham, Birmingham, GBR; 6 Medicine and Surgery, Sheikh Zayed Hospital Lahore, Lahore, PAK

**Keywords:** blunt trauma, cholecystitis, chest pain, intrathoracic gall bladder, diaphragmatic hernia

## Abstract

Herniation of abdominal contents through the diaphragm into the thoracic cavity can occur after blunt abdominal injury, resulting in a permanently acquired diaphragmatic hernia. Their clinical presentation is varied and non-specific, which can go unnoticed for a long duration.

A 27-year-old male presented with right upper quadrant pain and right-sided pleuritic chest pain for the past 20 days. His past medical history included high-impact blunt trauma a few years back. His workup revealed a right-sided diaphragmatic hernia through which the gallbladder had herniated into the thoracic cavity, along with liver and hepatic flexure of the colon. The gallbladder contained gallstones which were the cause of his symptoms. The patient was managed successfully with a laparotomy and repair of the diaphragmatic hernia and cholecystectomy.

After blunt abdominal trauma, right-sided diaphragmatic injury is less common because most of the trauma is absorbed by the liver, providing a protective effect. The sign and symptoms of acquired diaphragmatic hernia lack sensitivity and specificity, due to which many cases remain undiagnosed and are incidentally picked up on chest auscultation where bowel sounds are audible in the chest, and breath sounds on the affected side are absent, whereas patients have complaints of respiratory difficulty and recurrent pneumonia. Chest and abdominal imagining in the form of chest X-rays and abdominal ultrasound can help diagnose.

The case we present was a unique presentation of acquired right-sided diaphragmatic hernia resulting in herniation of the gallbladder in the right-sided chest and leading to acute cholecystitis. The treatment modality is surgical repair of the diaphragm. Any patient presenting with unusual symptoms of pneumonia or abdominal pain should be investigated, especially patients with a history of blunt abdominal trauma.

## Introduction

The abdominal and thoracic cavities are separated by a thin domed-shaped musculoaponeurotic barrier named diaphragm. The primary function of the diaphragm is to help in respiration; it acts as a barrier between two body cavities and respiratory muscle, which helps in the inspiration and expiration of gases during respiration essential to maintain life. Any penetrating injury can rapture the diaphragm to the abdominal or thoracic cavity. Although rare, diaphragmatic injuries are seen in surgical emergencies after a bullet or any penetrating injury [1, 2]. Another uncommon etiology is iatrogenic injury. Though very limited data is available, as it is very uncommon, but there is evidence in the literature of diaphragmatic injuries following major visceral resections like gastrectomy [3] or especially right-sided major liver resection [4].

The diaphragmatic injury was first recognized around 500 years ago in 1541 AD by Sennester, while Ridolfi’s first repair was done in 1886 [5]. The abdominal contents can herniate to the thoracic cavity through the diaphragm, known as a diaphragmatic hernia, which can be congenital or acquired. An acquired diaphragmatic hernia is usually present after penetrating injury or mal-repair of the diaphragm [6].

The incidence of diaphragmatic injury ranges from 1.1% to 3.9% after blunt trauma to a thoracic cavity or abdominal cavity. After the repair of a traumatic diaphragm, there is a 50% risk of developing a diaphragmatic hernia later in life. The signs and symptoms of such a hernia vary and are often nonspecific, like diffuse abdominal pain, so these diaphragmatic hernias can be missed during a routine clinical examination. When there is large rent causing the hernia of various organs, these hernias can present early in the course of the disease [7].

Acquired herniation through the diaphragm in adults mainly occurs after blunt abdominal or thoracic trauma, which may go unnoticed owing to nonspecific symptoms and remain undiagnosed for a long duration. In a large number of cases left side is involved, although presentation and underlying injury to the diaphragm can vary. The right side is involved in 10% of patients. In comparison, the left-side dome is involved in 90% of cases after injury and carries a high mortality rate due to damage to the heart and other vital organs lying in the left abdominal and thoracic cavity [8]. The protective effect of less injury on the right side is owed to the presence of the liver on the right side, which acts as a shock absorber during trauma, preventing damage to the diaphragm's right dome. But the mortality on the right side is even greater than left-sided injury because the injury required to cause rupture of the right diaphragm is enormous and damages the liver, pancreas, and intestine as well as pelvic organs making high mortality rates after right-sided diaphragm injury. If survived, it remains silent for a long duration and becomes evident incidentally during imaging for any other etiology or presents with complications of herniated abdominal viscera [9]. Usual presenting complaints include respiratory distress and non-responding cough, which is mistaken as recurrent pneumonia and being treated with excessive antibiotics. Still, no improvement in symptoms leads to extensive workup, and underlying pathology of diaphragmatic hernia is revealed, followed up with a referral to the surgeons. While some patients may present to surgical emergency with signs and symptoms of intestinal obstruction and workup for laparotomy reveals underlying diaphragmatic hernia on chest X-ray or ultrasound abdomen [9].

We here present a very unusual delayed presentation of a patient with a right diaphragmatic rupture following blunt trauma and herniation of his abdominal viscera, including gallbladder into the right hemithorax. The patient suffered from a sudden increase in his intra-abdominal pressure, caused by the direct impact of the fall of the tractor trolley over him due to slipping off the jack resulting in a rupture of the right dome of the diaphragm.

With this case report, we aim to educate surgeons about the unusual presentation of post-traumatic diaphragmatic hernia, concomitant pathology, and possible complications due to herniated organs.

## Case presentation

We are presenting this unusual case of a 27-year-old male patient who came to our surgical clinic complaining of right upper quadrant pain and right-sided pleuritic chest pain for the past 20 days. The patient quantified pain on a pain analog scale as 7/10. It was non-radiating and non-shifting pain with no specific characteristic. The pain was dull. It aggravated after spicy and fatty food intake and was relieved after taking oral painkillers or antacids. However, there were no other complaints of nausea, vomiting, dyspepsia, regurgitation, fever, or jaundice. There was no history of hematemesis or weight loss. At the time of admission, the patient was conscious and hemodynamically stable. His past medical history included blunt trauma nine years back. The patient was otherwise fit and well without any concomitant disease, had never had any surgery, and was a non-smoker. During examination absence of breath sounds in his right hemithorax and diffuse soft abdominal distension led to suspicion. Cardiac auscultation was unremarkable. Laboratory data showed a hemoglobin level of 14.6 g/dL, white blood cell count of 20,000/mm^3^, and platelet count of 356,000/mm^3^. His amylase, lipase, creatinine, and urea and electrolytes were within normal ranges.

**Figure 1:**
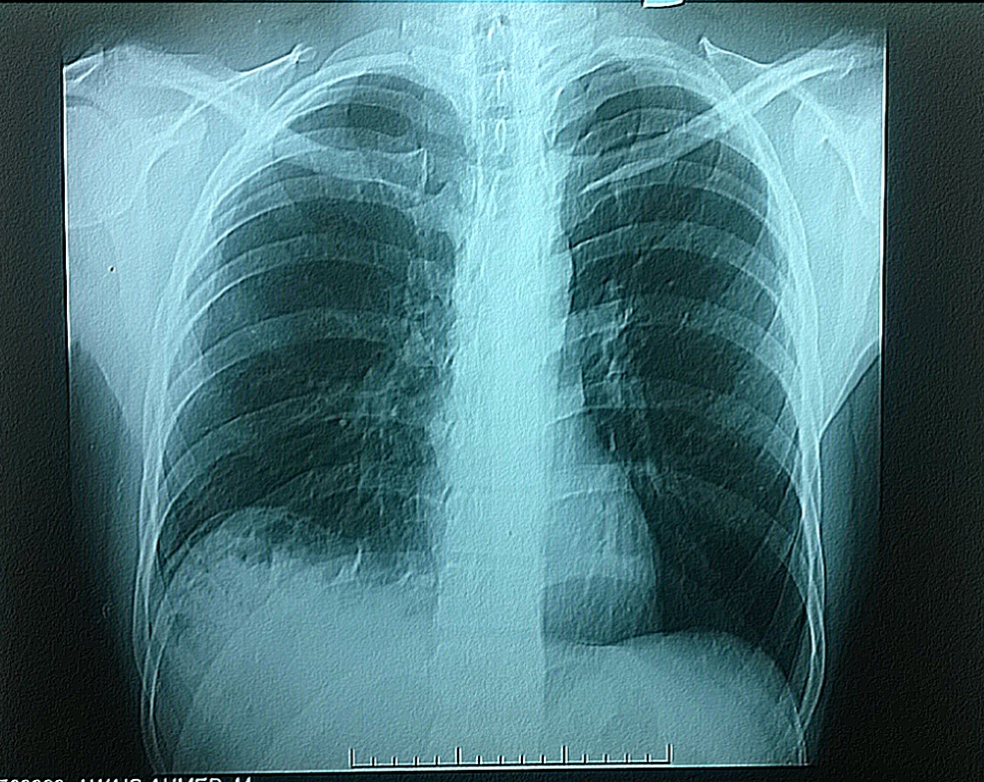
Preoperative CXR showing obliteration of the right dome of the diaphragm. CXR - Chest X-Ray

**Figure 2:**
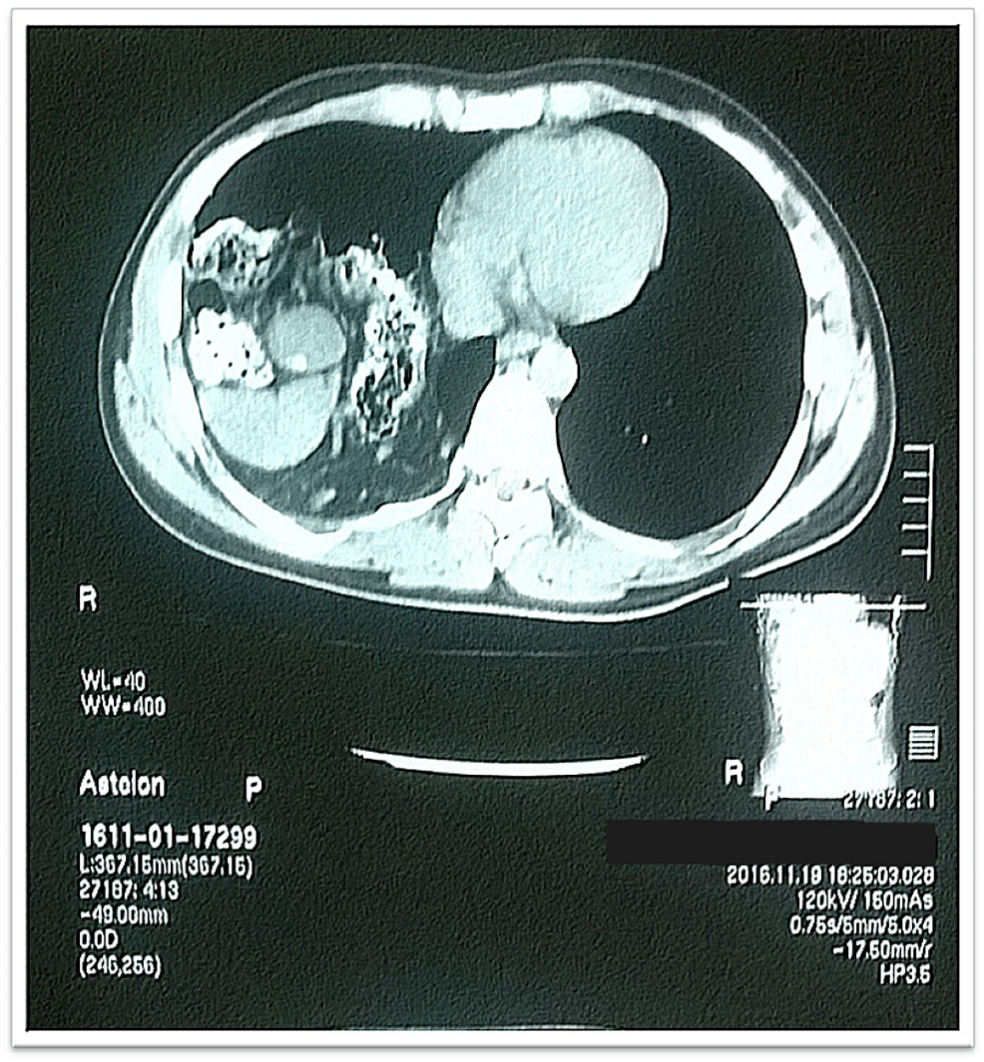
CT image showing herniated contents in the right hemithorax with a calculus in the gallbladder - Axial view CT - Computed Tomography

**Figure 3:**
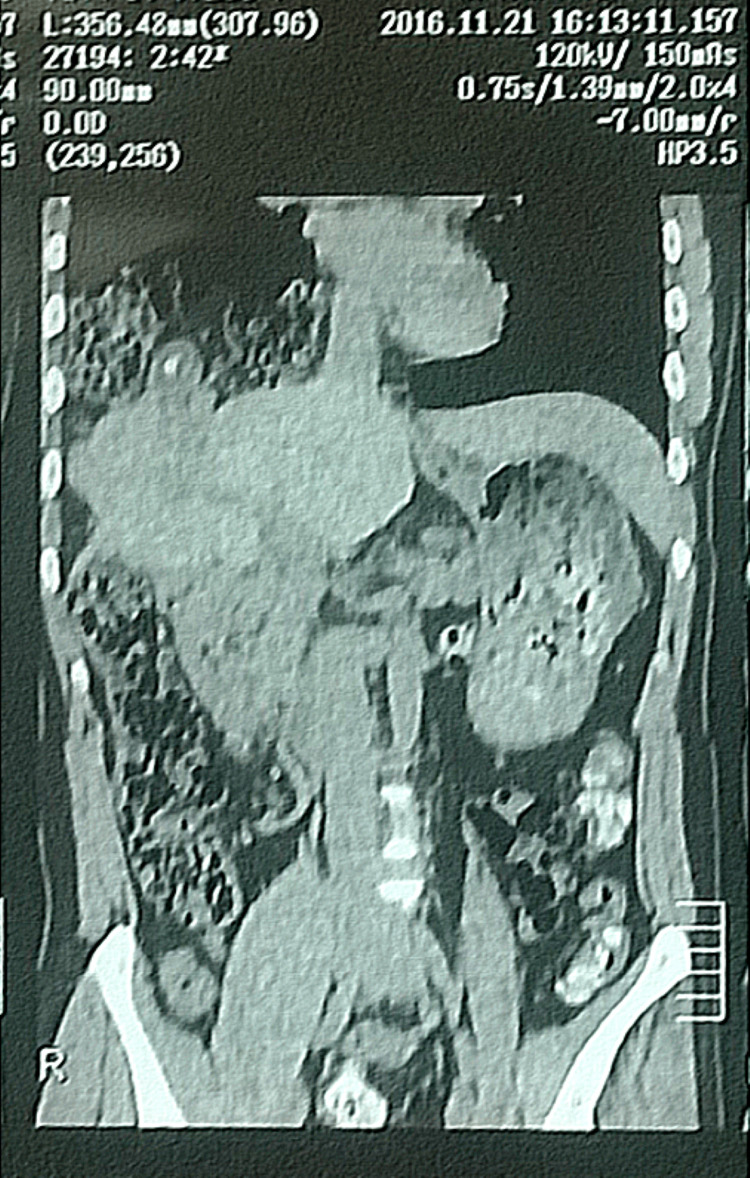
CT scan image showing herniated contents in the right hemithorax with a visible calculus in the gallbladder - Coronal view CT - Computed Tomography

His workup included a chest X-ray which showed loss of costophrenic angle and bowel loops in the right-sided chest cavity, as shown in Figure 1. A CT scan abdomen was done, which revealed a large right-sided diaphragmatic hernia with dimensions of 12x8cm through which the right lobe of the liver, gallbladder, part of the stomach, and hepatic flexure of the colon, and omentum were lying in the thoracic cavity, shown in Figure 2 and Figure 3. Gallbladder lumen showed multiple small calculi and features of cholecystitis.

**Figure 4:**
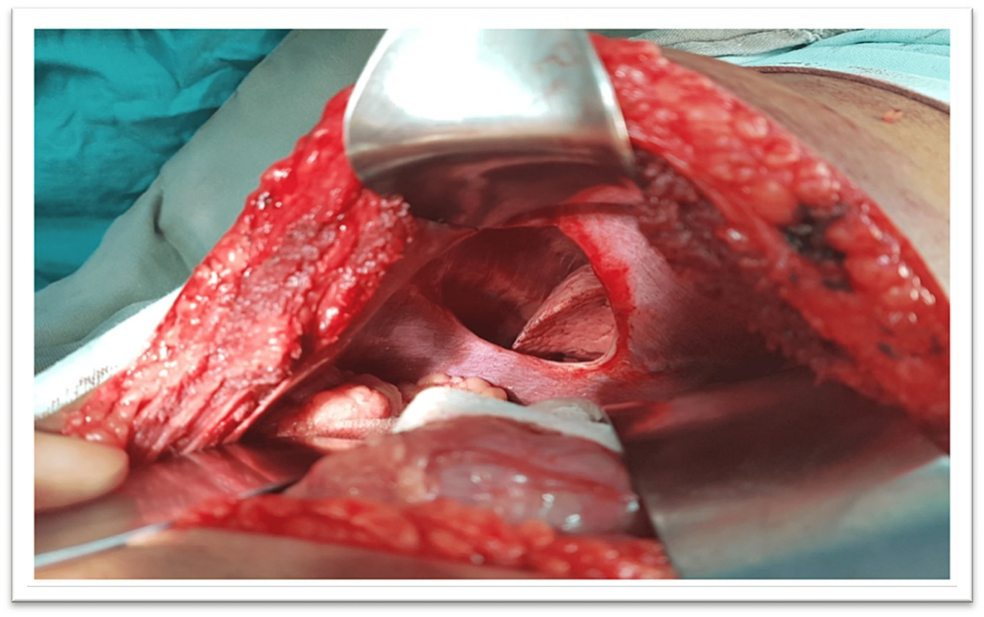
Right-sided diaphragmatic hernia, 8x10 cm defect in the right hemidiaphragm

**Figure 5:**
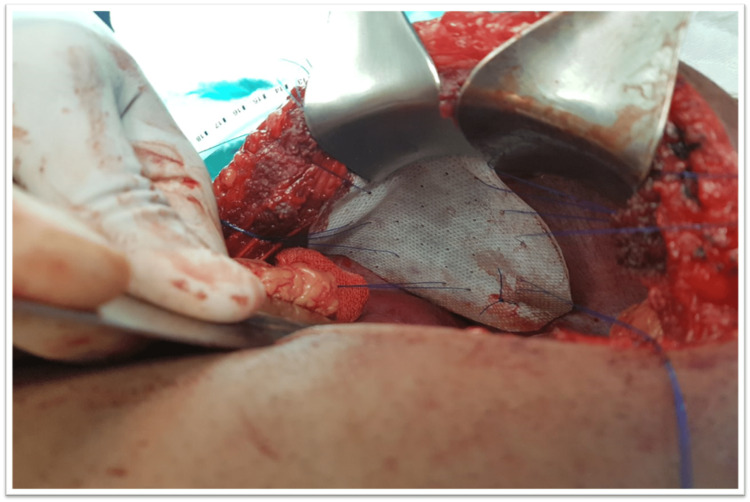
Dual mesh graft anchored with prolene sutures to cover the defect

After pre-operative anesthesia review and arrangement of blood products and taking written informed consent, laparotomy was performed. The patient also consented for the publication of this case report due to its unique presentation. During surgery, an 8x10cm defect was noted in the right diaphragm (see Figure 4) with gallbladder, hepatic flexure, partial right lobe of the liver, omentum, and antral part of the stomach pulled into the right-sided thoracic cavity. The gallbladder was distended, thick-walled with dense adhesions with the omentum, and contained multiple small calculi. All the abdominal organs herniating into the right hemithorax were placed back, and cholecystectomy was performed. The diaphragmatic defect was repaired with a dual mesh graft (Figure 5).

Post-operatively, the patient was kept in a high dependency unit, owing to the risk of intra-abdominal compartment syndrome. Post-operatively the patient was kept nil by mouth for 08 hours and was allowed initially oral sips and then liquids proceeding to a regular diet. Nasogastric tube was removed the same day. The patient was discharged on his 3rd postoperative day, and no complications were reported. The patient was called for a follow-up after one week and was stable. Another follow-up was done after a month, and the patient had no complications or concerns.

## Discussion

Blunt abdomen trauma in male patients is widespread because of road traffic accidents. These incidents lead to injury of right diaphragmatic injury in a small number of cases. The injuries can go unnoticed because they lack specific signs and symptoms. The patients can have very nonspecific chest or abdominal pain with no particular localization or radiation. In our case presentation, the patient presented with very unusual symptoms of chest pain on the right side, which was taken as pneumonia by a physician and was treated with antibiotics. Non-relieving pain resulted in further imaging and led to a diagnosis of intrathoracic cholecystitis and referral to surgeons for further management.

Similar cases of right-sided diaphragmatic hernias were reported by Das et al. [9], in which a 62-year-old male presented with combined pathology of acute cholecystitis and intrathoracic gallbladder along with a right-sided post-traumatic diaphragmatic hernia. History revealed that the cause of diaphragmatic hernia was a high-speed motor vehicle injury 24 years ago, resulting in spinal cord injury and weakness of both lower limbs later in life, presented with acute cholecystitis in the intrathoracic gallbladder. Ennis et al. [10] reported two cases of diaphragmatic hernia, one being treated as pneumonia just like our case and the second being treated for abdominal symptoms. But both of these cases were congenital diaphragmatic hernias, while our patient had acquired a diaphragmatic hernia after traumatic injury years ago and remained unnoticed.

Tahiri et al. [11] reported a case of a 57-year-old male presenting with respiratory symptoms leading to the diagnosis of prolapsed gallbladder and other abdominal organs through the right dome of the diaphragm. The patient had acute cholecystitis, and a cholecystectomy was done. There was a history of blunt abdominal trauma seven years back, resulting in a right-sided acquired diaphragmatic hernia. Later, the patient was operated on, abdominal organs were pushed back to the abdominal cavity, and diaphragmatic repair was done using a prolene mesh graft.

Ioannidis et al. reported a female having blunt abdominal trauma resulting in a right-sided diaphragmatic injury and protrusion of the right lobe of the liver in the thoracic cavity along with a fracture of the right lower ribs [12]. Other unusual presentations may include other abdominal organs in the thorax, as seen in a case report of Daha et al. in which a 24-year-old female with a right thoracic kidney with Bochdalek hernia was diagnosed incidentally [13]. Korkut et al. reported a 28-year-old female who presented with bowel obstruction and subsequently was found to have bowel necrosis at the time of emergency surgery, where small bowel and transverse colon herniated into the right-sided diaphragmatic hernia after a right laparoscopic hepatectomy due to a hepatic alveolar cyst 20 months ago [14].

The purpose of this case report and discussion is to create evidence-based awareness among physicians to look for multiple pathologies in a single patient so that accurate and complete diagnoses can be made, resulting in the optimal care of patients.

Although laparoscopic or thoracoscopic management of such patients may become prevalent with increasing experience, the open approach and simple repair remain the mainstays of control. Preoperatively, all viscera should be inspected for any signs of injury. Patients should be managed in high-dependency units as they are at risk of abdominal compartment syndrome, and if that happens, they can deteriorate quickly.

## Conclusions

The case we presented was a unique presentation of acquired right-sided diaphragmatic hernia resulting in herniation of the gallbladder in the right-sided chest and leading to acute cholecystitis. The treatment modality is surgical repair of the diaphragm with cholecystectomy. Any patient presenting with unusual symptoms of pneumonia or abdominal pain should be investigated, mainly in patients having a history of blunt abdominal trauma.
